# Clades of huge phages from across Earth’s ecosystems

**DOI:** 10.1038/s41586-020-2007-4

**Published:** 2020-02-12

**Authors:** Basem Al-Shayeb, Rohan Sachdeva, Lin-Xing Chen, Fred Ward, Patrick Munk, Audra Devoto, Cindy J. Castelle, Matthew R. Olm, Keith Bouma-Gregson, Yuki Amano, Christine He, Raphaël Méheust, Brandon Brooks, Alex Thomas, Adi Lavy, Paula Matheus-Carnevali, Christine Sun, Daniela S. A. Goltsman, Mikayla A. Borton, Allison Sharrar, Alexander L. Jaffe, Tara C. Nelson, Rose Kantor, Ray Keren, Katherine R. Lane, Ibrahim F. Farag, Shufei Lei, Kari Finstad, Ronald Amundson, Karthik Anantharaman, Jinglie Zhou, Alexander J. Probst, Mary E. Power, Susannah G. Tringe, Wen-Jun Li, Kelly Wrighton, Sue Harrison, Michael Morowitz, David A. Relman, Jennifer A. Doudna, Anne-Catherine Lehours, Lesley Warren, Jamie H. D. Cate, Joanne M. Santini, Jillian F. Banfield

**Affiliations:** 1 Innovative Genomics Institute, University of California Berkeley, Berkeley, CA USA; 2 National Food Institute, Technical University of Denmark, Kongens Lyngby, Denmark; 3 Earth and Planetary Science, University of California Berkeley, Berkeley, CA USA; 4 Nuclear Fuel Cycle Engineering Laboratories, Japan Atomic Energy Agency, Tokai-mura, Japan; 5 Department of Microbiology & Immunology, Stanford University, Stanford, CA USA; 6 Department of Soil and Crop Sciences, Colorado State University, Fort Collins, CO USA; 7 Department of Civil and Mineral Engineering, University of Toronto, Toronto, Ontario Canada; 8 Environmental Science, Policy and Management, University of California Berkeley, Berkeley, CA USA; 9 DOE Joint Genome Institute, Berkeley, CA USA; 10 Integrative Biology, University of California Berkeley, Berkeley, CA USA; 11 School of Life Sciences, Sun Yat-Sen University, Guangzhou, China; 12 Centre for Bioprocess Engineering Research, University of Cape Town, Cape Town, South Africa; 13 Department of Surgery, University of Pittsburgh School of Medicine, Pittsburgh, PA USA; 14 Laboratoire Microorganismes: Génome et Environnement, Université Clermont Auvergne, CNRS, Clermont-Ferrand, France; 15 Institute of Structural and Molecular Biology, University College London, London, UK; 16 School of Earth Sciences, University of Melbourne, Melbourne, Victoria Australia

**Keywords:** Bacteriophages, Bacteriophages, Environmental microbiology, Environmental microbiology, Metagenomics

## Abstract

Bacteriophages typically have small genomes^1^ and depend on their bacterial hosts for replication^2^. Here we sequenced DNA from diverse ecosystems and found hundreds of phage genomes with lengths of more than 200 kilobases (kb), including a genome of 735 kb, which is—to our knowledge—the largest phage genome to be described to date. Thirty-five genomes were manually curated to completion (circular and no gaps). Expanded genetic repertoires include diverse and previously undescribed CRISPR–Cas systems, transfer RNAs (tRNAs), tRNA synthetases, tRNA-modification enzymes, translation-initiation and elongation factors, and ribosomal proteins. The CRISPR–Cas systems of phages have the capacity to silence host transcription factors and translational genes, potentially as part of a larger interaction network that intercepts translation to redirect biosynthesis to phage-encoded functions. In addition, some phages may repurpose bacterial CRISPR–Cas systems to eliminate competing phages. We phylogenetically define the major clades of huge phages from human and other animal microbiomes, as well as from oceans, lakes, sediments, soils and the built environment. We conclude that the large gene inventories of huge phages reflect a conserved biological strategy, and that the phages are distributed across a broad bacterial host range and across Earth’s ecosystems.

## Abstract (web summary)

Genomic analyses of major clades of huge phages sampled from across Earth’s ecosystems show that they have diverse genetic inventories, including a variety of CRISPR–Cas systems and translation-relevant genes.

## Main

**Extended Data Fig. 1:**
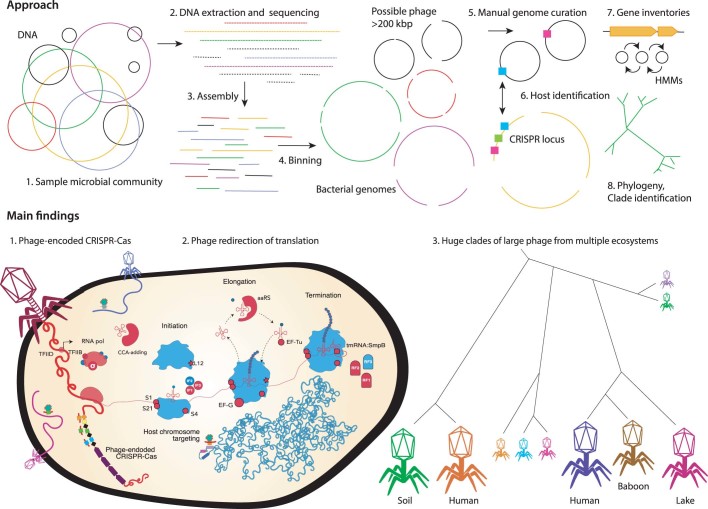
Graphical abstract describing the approach and main findings of this study. aaRS, aminoacyl-tRNA synthetase; CCA-adding, tRNA nucleotidyltransferase; EF, elongation factor; IF, initiation factor; PDF, peptide deformylase; QueC/D/F, queuosine synthesis and tRNA modification; RF, release factor; RNA Pol, RNA polymerase; RRF, ribosome recycling factor; TFIIB, transcription factor II B.

Phages—viruses that infect bacteria—are considered distinct from cellular life owing to their inability to carry out most biological processes required for reproduction. They are agents of ecosystem change because they prey on specific bacterial populations, mediate lateral gene transfer, alter host metabolism and redistribute bacterially derived compounds through cell lysis^2–4^. They spread antibiotic resistance^5^ and disperse pathogenicity factors that cause disease in humans and animals^6,7^. Most knowledge about phages is based on laboratory-studied examples, the vast majority of which have genomes that are a few tens of kb in length. Widely used isolation-based methods select against large phage particles, and they can be excluded from phage concentrates obtained by passage through 100-nm or 200-nm filters^1^. In 2017, only 93 isolated phages with genomes that were more than 200 kb in length were published^1^. Sequencing of whole-community DNA can uncover phage-derived fragments; however, large genomes can still escape detection owing to fragmentation^8^. A new clade of human- and animal-associated megaphages was recently described on the basis of genomes that were manually curated to completion from metagenomic datasets^9^. This finding prompted us to carry out a more-comprehensive analysis of microbial communities to evaluate the prevalence, diversity and ecosystem distribution of phages with large genomes. Previously, phages with genomes of more than 200 kb have been referred to as ‘jumbophages’^1^ or, in the case of phages with genomes of more than 500 kb, as megaphages^9^. As the set reconstructed here span both size ranges we refer to them simply as ‘huge phages’. A graphical abstract provides an overview of our approach and main findings (Extended Data Fig. 1). This study expands our understanding of phage biodiversity and reveals the wide variety of ecosystems in which phages have genomes with sizes that rival those of small-celled bacteria^10–12^. We postulate that these phages have evolved a distinct ‘life’ strategy that involves extensive interception and augmentation of host biology while they replicate their huge genomes.

## Ecosystem sampling

**Extended Data Fig. 2:**
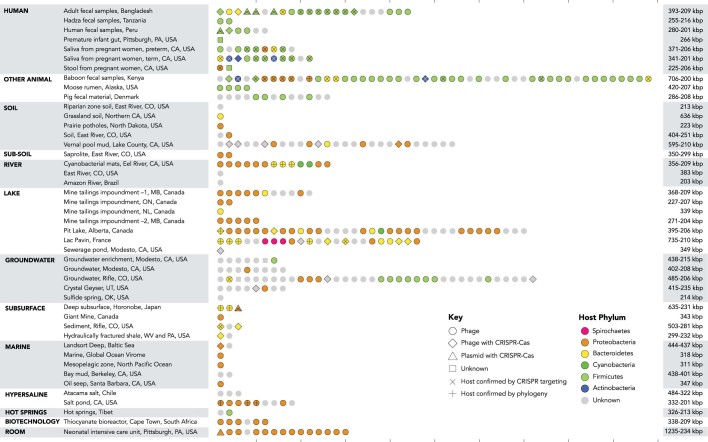
Ecosystems with phage genomes and plasmid-like sequences of more than 200 kb. Genomes grouped by sampling-site type. Each box represents a phage genome or plasmid-like sequence, and boxes are horizontally arranged in order of decreasing genome size. The size range for each site type is listed to the right. Colours indicate putative host phylum on the basis of genome taxonomic profile, with confirmation by CRISPR spacer targeting (X) or rpS21 (+).

Metagenomic datasets were acquired from human faecal and oral samples, faecal samples from other animals, freshwater lakes and rivers, marine ecosystems, sediments, hot springs, soils, deep subsurface habitats and the built environment (Extended Data Fig. 2). Genome sequences that were clearly not bacterial, archaeal, archaeal virus, eukaryotic or eukaryotic virus were classified as phage, plasmid-like or mobile genetic elements of uncertain nature on the basis of their gene inventories (Supplementary Information). De novo assembled fragments close to or more than 200 kb in length were tested for circularization and a subset was selected for manual verification and curation to completion (Methods).

## Genome sizes and basic features

**Extended Data Fig. 3:**
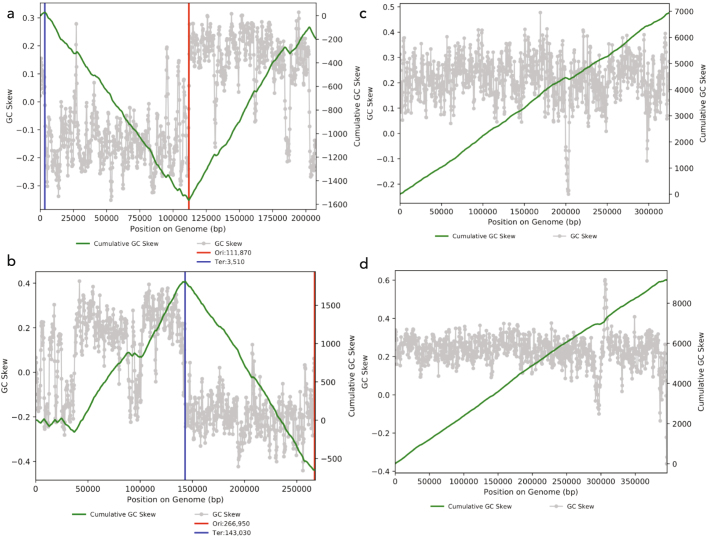
Examples of phage genomes that display GC skew indicative of bidirectional replication. **a**, **b**, Example phage genomes with GC skew patterns that are strongly indicative of bidirectional replication (origin-to-terminus) that is typically found in bacteria (however, the origin may not correspond to the start of the genome). **c**, **d**, Phage genomes with GC skew patterns that are suggestive of unidirectional patterns.

We reconstructed 351 phage sequences, 6 plasmid-like sequences and 4 sequences of unknown classification (Extended Data Fig. 2). We excluded additional sequences that were inferred to be plasmids (Methods), retaining only those that encoded CRISPR–Cas loci. We included 3 phage sequences of ≤200 kb in length owing to the presence of CRISPR–Cas loci. Consistent with the classification as phages, we identified a wide variety of phage-relevant genes, including those involved in lysis and encoding structural proteins, and documented other expected genomic features of phages (Supplementary Information). Some predicted proteins were large, up to 7,694 amino acids in length; some were tentatively annotated as structural proteins. In total, 175 phage sequences were circularized and 35 were manually curated to completion, in some cases by resolving complex repeat regions, revealing their encoded proteins (Methods and Supplementary Table 1). The remaining genomes are probably incomplete, although some may be complete, but linear. Approximately 30% of genomes show clear GC skew indicative of bidirectional replication and 30% have patterns indicative of unidirectional replication^13^ (Extended Data Fig. 3 and Supplementary Information).

**Fig. 1:**
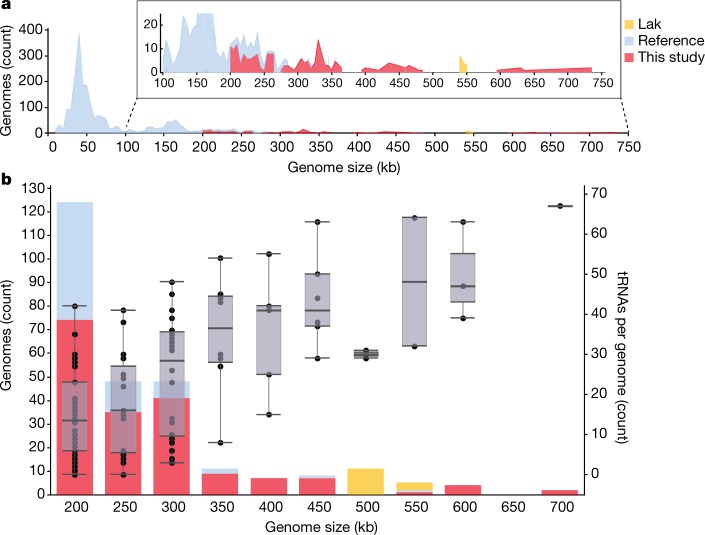
Distribution of the genome sizes and tRNAs of phages. **a**, Size distribution of circularized bacteriophage genomes from this study, Lak megaphage genomes reported recently for a subset of the same samples^9^ and reference sources. Reference genomes were collected from all complete RefSeq r92 dsDNA genomes and non-artefactual assemblies with lengths of more than 200 kb from a previous study^14^. **b**, Histogram of the genome size distribution of phages with genomes of more than 200 kb from this study, Lak and reference genomes. Box-and-whisker plot of tRNA counts per genome from this study and Lak phages as a function of genome size (Spearman’s *ρ* = 0.61, *P* = 4.5 × 10^−22^, *n* = 201 individual phage genomes). The middle line for each box marks the median tRNA count for each size bin, the box marks the interquartile range, and the whiskers represent the maximum and minimum.

Our 4 largest complete, manually curated and circularized phage genomes are 634, 636, 642 and 735 kb in length and are—to our knowledge—the largest phage genomes reported to date. The largest previously reported circularized phage genome was 596 kb in length^14^. The same previous study also reported a circularized genome of 630 kb in length; however, this is an assembly artefact (Supplementary Information). The problem of concatenation artefacts was sufficiently prominent in IMG/VR^15^ that we did not include these data in further analyses. We used both complete and circularized genomes from our study and published phage genomes to produce an updated view of the distribution of phage genome sizes (Methods). Without the huge phages reported here, the median genome size for complete phages is around 52 kb (Fig. 1a). Thus, the sequences reported here substantially expand the inventory of phages with unusually large genomes (Fig. 1b).

**Extended Data Fig. 4:**
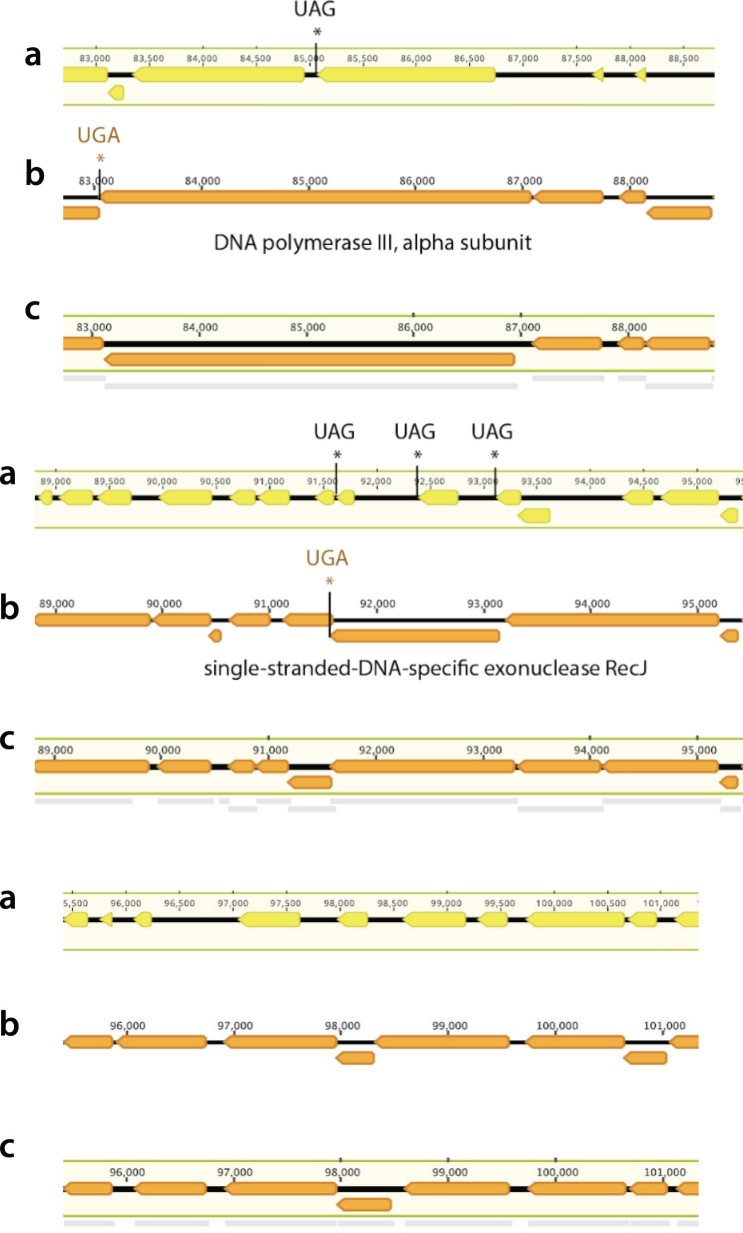
Example of the alternative coding of phages. Comparisons of gene predictions for a region with genes of clearly predicted function in M05_PHAGE_COMPLETE_32_3. **a**, The standard (code 11) genetic code. **b**, Both TAG and TAA repurposed (code 6). **c**, TAG repurposed (code 16). Overall, analysis of well-annotated genes supported code 16 as the best choice (TAG to X, as X could not be clearly resolved on the basis of sequence alignments with related proteins).

Some of our reported genomes have a very low coding density (9 genomes have densities of less than 78%) (Supplementary Information), probably owing to the use of a genetic code that is different from the standard code (Methods). This phenomenon has been rarely noted in phages, but has previously been reported for the Lak phage^9^ and in a previous study^16^. In the current study, some genomes (mostly those that are associated with humans and/or animals) appear to have reassigned the UAG (amber) stop codon to encode an amino acid (Extended Data Fig. 4 and Supplementary Information).

In only one case, we identified a sequence of more than 200 kb that was classified as a prophage on the basis of the transition into a flanking bacterial genome sequence. However, around half of the genomes were not circularized, so their potential integration as a prophage cannot be ruled out. The presence of integrases in some genomes is suggestive of a temperate lifestyle under some conditions.

## Hosts, diversity and distribution

**Fig. 2:**
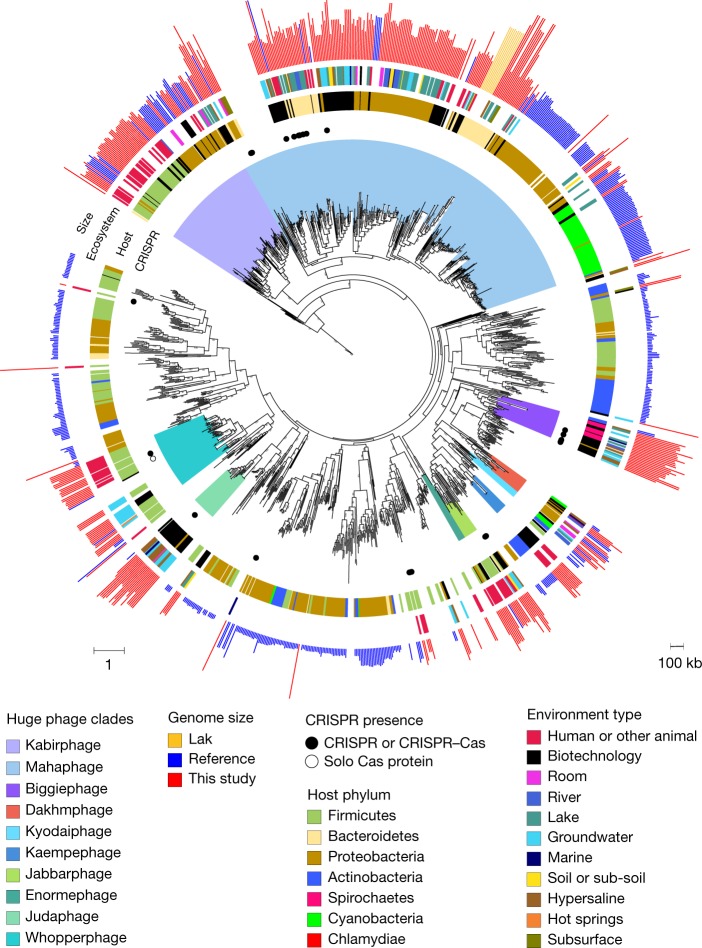
Phylogenetic reconstruction of the evolutionary history of huge phages. The phylogeny of phages was reconstructed using large terminase sequences from this study (*n* = 397) and similar matches from all RefSeq r92 proteins (*n* = 532). The tree also includes large terminase sequences from complete RefSeq phage, the Lak megaphage clade^9^ (*n* = 9) and non-artefactual phage genomes that are more than 200 kb, from a previous study^14^. Huge phage clades identified in this study were independently corroborated with a phylogenetic reconstruction of major capsid protein (MCP) genes (Extended Data Fig. 5a) and protein clustering (Extended Data Fig. 5b). The tree was rooted using eukaryotic herpesvirus terminases (*n* = 7). The inner to outer rings display the presence of CRISPR–Cas in this study, host phylum, environmental sampling type and genome size. Host phylum and genome size were not included for RefSeq protein database matches for which the sequence may be from an integrated prophage or part of organismal genome projects. Scale bars show the number of substitutions per site (left) and number of base pairs (right).

**Extended Data Fig. 5:**
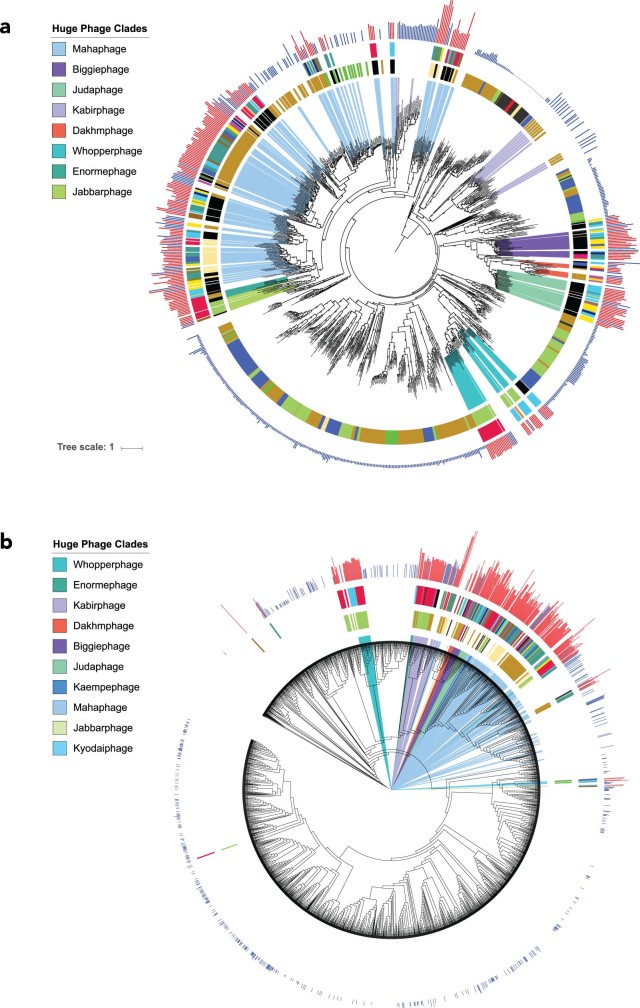
Phylogenetic and protein-cluster relationships between phages. **a**, The phylogenetic tree of phages based on the MCPs. The outer ring shows genome length; bars in red indicate genomes reconstructed and reported in this study and bars in blue indicate database genomes. The next ring indicates the environment of origin. The inner ring indicates the phylum of the host (black indicates unknown). Superimposed colours indicate named clades that consist of huge phages that were identified in the terminase tree. Colours are as in Fig. 2. **b**, Hierarchical clustering dendrogram of phage genomes based on the Jaccard distance between the presence or absence profiles of protein families, performed using an average linkage method. The outermost ring shows phage genome length, the next ring shows the environment of origin, then predicted phylum affiliation of bacterial hosts. Superimposed colours indicate named clades that consist of huge phages that were identified in the terminase tree. Colours are as in Fig. 2. The clustering supports the phylogenetic analyses shown in **a** and Fig. 2.

An intriguing question relates to the evolutionary history of phages with huge genomes; namely, whether they are the result of recent genome expansion within clades of normal-sized phages or whether a large inventory of genes is an established, persistent strategy. To investigate this, we constructed phylogenetic trees for large terminase subunit proteins (Fig. 2) and major capsid proteins (Extended Data Fig. 5a) using sequences from public databases as a context (Methods). Many of the sequences from our phage genomes cluster together with high bootstrap support, thus defining clades. Analysis of the genome size information for database sequences shows that the public sequences that fall into these clades are from phages with genomes of at least 120 kb in length. The largest clade, referred to here as Mahaphage (Maha being Sanskrit for huge), includes all of our largest genomes as well as the 540–552 kb Lak genomes from human and animal microbiomes^9^. We identified nine other clusters of large phages, and refer to them using the words for ‘huge’ in the languages of some authors of this paper. We acknowledge that the detailed tree topologies for different genes and datasets vary slightly; however, the clustering is broadly supported by protein family and capsid analyses (Extended Data Fig. 5a, b). The fact that large phages are consistently grouped together into clades establishes that a large genome size is a relatively stable trait. Within each clade, phages were sampled from a wide variety of environment types (Fig. 2), indicating the diversification of these huge phages and their hosts across ecosystems. We also examined the environmental distribution of phages that are so closely related that their genomes can be aligned and we found 20 cases in which the phages occur in at least 2 distinct cohorts or habitat types (Supplementary Table 2).

To determine the extent to which bacterial host phylogeny correlates with phage clades, we identified some phage hosts using CRISPR spacer targeting from bacteria in the same or related samples and phylogenies of normally host-associated phage genes (see below, Supplementary Table 3). We also tested the predictive value of bacterial taxonomic affiliations of the phage gene inventories (Methods) and found that in every case, CRISPR spacer targeting and phylogeny agreed with phylum-level taxonomic profiles. We therefore used taxonomic profiles to predict the bacterial host phylum for many phages (Supplementary Table 4). The results establish the importance of Firmicutes and Proteobacteria as hosts (Extended Data Fig. 2) (*P* = 2.5 × 10^−5^, *n* = 74, *W* = 606; one-sided Wilcoxon signed-rank test). The higher prevalence of Firmicutes-infecting huge phages in the human and animal gut compared with other environments reflects the potential host compositions of the microbiomes (*P* = 9.3 × 10^−7^, *n* = 37, *U* = 238; one-sided Mann–Whitney *U*-test). Notably, the 5 genomes that were more than 634 kb in length were all from phages that were predicted to replicate in Bacteroidetes, as do Lak phages^9^, and all cluster within the Mahaphage clade. Overall, phages that grouped together phylogenetically are predicted to replicate in bacteria of the same phylum (Fig. 2).

## Metabolism, transcription and translation

The phage genomes encode proteins that are predicted to localize to the bacterial membrane or cell surface. These may affect the susceptibility of the host to infection by other phages (Supplementary Table 5 and Supplementary Information). We identified almost all of the previously reported categories of genes that have been suggested to augment host metabolism (Supplementary Information). Many phages have genes involved in the de novo biosynthesis of purines and pyrimidines, and the interconversion of nucleic and ribonucleic acids and nucleotide phosphorylation states. These gene sets are intriguingly similar to those of bacteria with very small cells and putative symbiotic lifestyles^10^ (Supplementary Table 5).

Notably, many phages have genes with predicted functions in transcription and translation (Supplementary Table 6). Complete phage genomes encode up to 67 tRNAs, with sequences that are distinct from those of their hosts (Supplementary Table 7). Generally, the number of tRNAs per genome increases with genome length (Fig. 1) (Spearman’s *ρ* = 0.61, *P* = 4.5 × 10^−22^, *n* = 201). Huge phages have up to 15 tRNA synthetases per genome (Supplementary Table 7), which are also distinct from but related to those of their hosts (Extended Data Fig. 7a and Supplementary Information). Phages may use these proteins to charge their own tRNA variants with host-derived amino acids. A subset of genomes has genes for tRNA modification and ligation of tRNAs cleaved by host defenses.

**Fig. 3:**
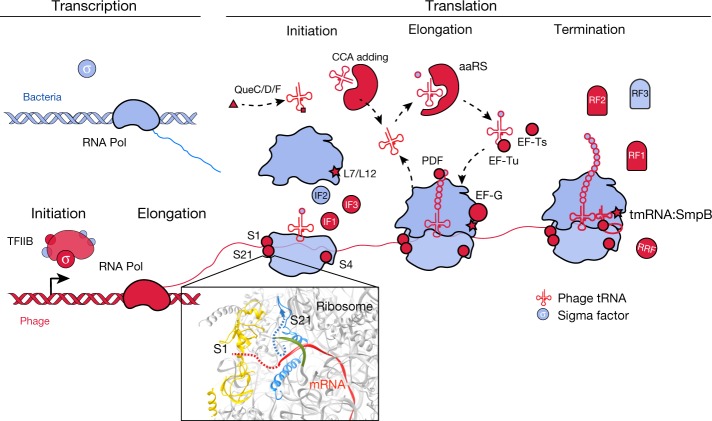
A model for phage interception and redirection of host translational systems. Potential mechanisms for how phage-encoded capacities could function to redirect the translational system of the host to produce phage proteins (bacterial components in blue, phage proteins in red). No huge phage encodes all translation-related genes, but many have tRNAs and tRNA synthetases (Supplementary Table 6). Phage proteins with up to six ribosomal protein S1 domains occur in a few genomes. The S1 binds to mRNA to bring it into the site on the ribosome where it is decoded^39^. Phage ribosomal protein S21 might promote translation initiation of phage mRNAs, and many sequences have N-terminal extensions that may be involved in binding RNA (dashed blue line in ribosome insert (RCSB Protein Data Bank (PDB) code: 6BU8^40^)), analysed with UCSF Chimera^41^. Many other proteins of the translational apparatus that belong to all steps of the translation cycle are encoded by huge phages. aaRS, aminoacyl-tRNA synthetase; CCA-adding, tRNA nucleotidyltransferase; EF, elongation factor; IF, initiation factor; PDF, peptide deformylase; QueC/D/F, queuosine synthesis and tRNA modification; RF, release factor; RNA Pol, RNA polymerase; RRF, ribosome recycling factor; TFIIB, transcription factor II B.

Many phages carry genes that are implicated in the interception and redirection of host translation. These genes include the initiation factors IF1 and IF3, as well as ribosomal proteins S4, S1, S21 and L7/L12 (ribosomal proteins were only recently reported in phages^17^ (Fig. 3)). Both rpS1 and rpS21 are important for translation initiation in bacteria^18–20^, making them likely to be useful for the hijacking of host ribosomes. Further analysis of rpS21 proteins revealed N-terminal extensions that were rich in basic and aromatic residues important for RNA binding. We predict that these phage ribosomal proteins substitute for host proteins^17^, and their extensions assist in competitive ribosome binding or preferential initiation of phage mRNAs.

Because rpS1 is often studied in the context of Shine–Dalgarno sequence recognition by the ribosome^19,20^, we predicted the ribosomal binding sites for each phage genome (Methods). Whereas most phages have canonical Shine–Dalgarno sequences, huge phages from this study that carry possible rpS1s rarely have identifiable Shine–Dalgarno sequences (Supplementary Information and Supplementary Table 8). It is difficult to confirm ‘true’ rpS1 proteins owing to the ubiquity of the S1 domain, but this correlation with non-canonical Shine–Dalgarno sequences suggests a role in translation initiation, either on or off the ribosome.

Although assuming control of initiation may be the most logical step for the redirection of host translation by the phage, improving the efficiency of elongation and termination is necessary for robust infection and replication. Accordingly, we found many genes associated with the later steps of translation in phage genomes. These include elongation factors G, Tu and Ts, rpL7/12 and the processing enzyme peptide deformylase (Fig. 3), which has previously been reported in phage genomes^21^. We hypothesize that phage-encoded elongation factors maintain the overall translation efficiency during infection, much like the previously predicted role of peptide deformylase in sustaining translation of the necessary photosynthetic proteins of the host^21^. Translation termination factors are also represented in our huge phage genomes, including release factor 1 and 2, ribosome recycling factor, as well as transfer messenger RNAs (tmRNAs) and small protein B (SmpB), which rescue ribosomes stalled on damaged transcripts and trigger the degradation of aberrant proteins. These tmRNAs are also used by phages to sense the physiological state of host cells and can induce lysis when the number of stalled ribosomes in the host is high^22^. Notably, some large putative plasmids have analogous suites of translationrelevant genes (Supplementary Table 5).

## CRISPR–Cas-mediated interactions

We identified most major types of CRISPR–Cas systems in phages, including Cas9-based type II, the recently described type V-I^23^, new variants of the type V-U systems^24^ and new subtypes of the type V-F system^25^ (Extended Data Fig. 8). The class II systems (types II and V) have not previously been reported in phages. Most phage effector nucleases (for interference) have conserved catalytic residues, implying that they are functional.

**Fig. 4:**
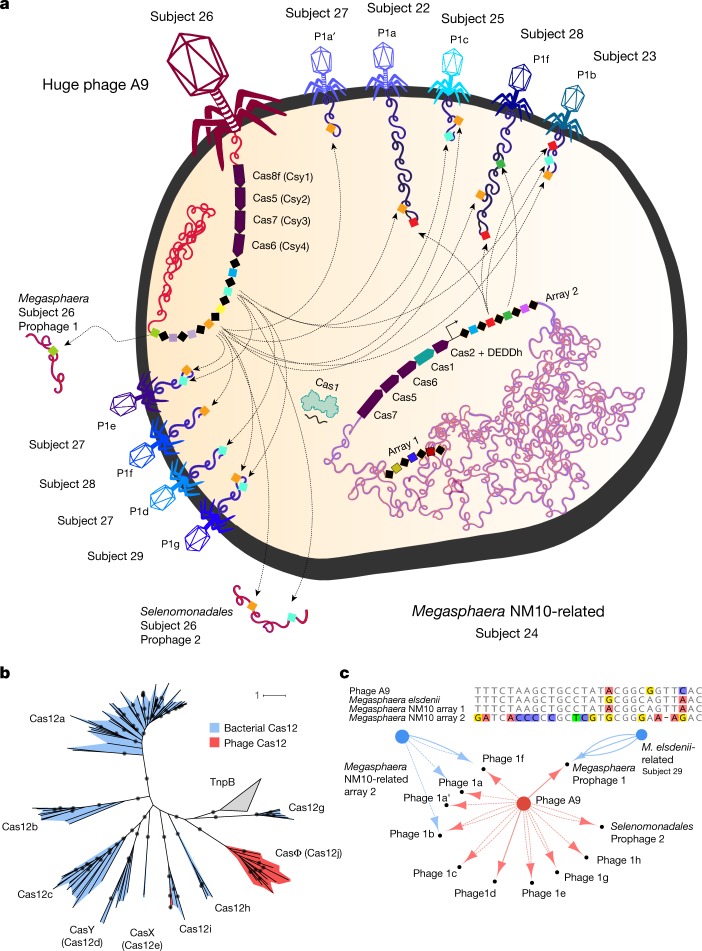
Phage and bacterial CRISPR-interaction dynamics. **a**, Cell diagram of bacterium–phage and phage–phage interactions that involve CRISPR targeting during superinfection. Arrows indicate CRISPR–Cas targeting of the prophage and phage genomes. Phage names indicate related groups delineated by whole-genome alignment. We only included CRISPR interactions from samples of subjects of the same human cohort. **b**, Maximum likelihood phylogenetic tree of Cas12 subtypes a–i. Phage-encoded Cas12i and CasΦ, the new effector, are outlined in red, with bacteria-encoded proteins in blue. Bootstrap values >90 are shown on the branches (circles). Cas14 and type V-U trees are provided separately (Supplementary Fig. 11). Scale bars indicates the number of substitutions per site. **c**, Top, alignment of the consensus repeats from the A9 phage array and predicted host bacterial arrays. Bottom, interaction network showing the targeting of bacteria-encoded (blue) and phage-encoded (red) CRISPR spacers. The number of edges indicate the number of spacers from the array with targets to the smaller node. Solid edges denote spacer targets with no or one mismatch, and dashed edges denote two to three mismatches (to account for degeneration in old-end phage spacers, diversity in different subjects or phage mutation to avoid targeting).

In contrast to the well-described case of a phage with a CRISPR system^26^, almost all phage CRISPR systems lack spacer acquisition machinery (Cas1, Cas2 and Cas4) and many lack recognizable genes for interference (Extended Data Fig. 9 and Supplementary Table 1). For example, two related phages have a type I-C variant system that lacks Cas1 and Cas2 and have a helicase protein instead of Cas3. These phages also have a second system that contains a new candidate type V effector protein, CasΦ (Cas12j), which is approximately 750 amino acids in length (Fig. 4 and Supplementary Table 1), which occurs proximal to CRISPR arrays.

In some cases, phages that lack genes for interference and spacer integration have similar CRISPR repeats as their hosts (Fig. 4c) and may therefore use the Cas proteins of the host. Alternatively, systems that lack an effector nuclease may repress the transcription of the target sequences without cleavage^27,28^. Additionally, spacer-repeat guide RNAs may have an RNA-interference-like mechanism to silence host CRISPR systems or nucleic acids to which they can hybridize. The phage-encoded CRISPR arrays are often compact (median, six repeats per array) (Extended Data Fig. 10). This range is substantially smaller than typically found in prokaryotic genomes (mean of 41 repeats for class I systems)^29^. Some phage spacers target core structural and regulatory genes of other phages (Fig. 4c and Supplementary Table 10). Thus, phages apparently augment the immune arsenal of their hosts to prevent infection by competing phages.

Some phage-encoded CRISPR loci have spacers that target bacteria in the same sample or in a sample from the same study. We suppose that the targeted bacteria are the hosts for these phages, an inference supported by other host prediction analyses (Supplementary Table 4). Some loci with bacterial chromosome-targeting spacers encode Cas proteins that could cleave the host chromosome, whereas others do not. The targeting of host genes could disable or alter their regulation, which may be advantageous during the phage infection cycle. Some phage CRISPR spacers target bacterial intergenic regions, possibly interfering with genome regulation by blocking promoters or silencing non-coding RNAs.

Notable examples of CRISPR targeting of bacterial chromosomes involve transcription and translation genes. For instance, one phage targets a σ^70^ transcription factor gene in the genome of its host and encodes its own σ^70^ (Supplementary Information). Some huge phage genomes encode anti-sigma factor-like proteins (AsiA), consistent with previous reports of σ^70^ hijacking by phages with AsiA^30^. In another example, a phage spacer targets the host glycyl tRNA synthetase, but the Cas14 effector lacks one of the required catalytic residues for cleavage, suggesting a role in repression (as a ‘dCas14’), rather than in cleavage (Supplementary Information).

Notably, we found no evidence of host-encoded spacers that target any CRISPR-bearing phages. However, phage CRISPR targeting of other phages that are also targeted by bacterial CRISPR (Fig. 4c) suggested phage–host associations that were broadly confirmed by the phage taxonomic profile (Supplementary Table 4).

Some large *Pseudomonas-*infecting phages encode anti-CRISPRs^31,32^ (Acrs) and proteins that assemble a nucleus-like compartment that segregates their replicating genomes from host-defence and other bacterial systems^33^. We identified proteins encoded in huge phage genomes that cluster with AcrVA5, AcrVA2, AcrIIA7 and AcrIIA11 and may function as Acrs. We also identified tubulin homologues (PhuZ) and proteins (Supplementary Information) that create a proteinaceous phage ‘nucleus’^34^. The phage nucleus was recently shown to protect the phage genome against host defence by physically blocking degradation by CRISPR–Cas systems^35^.

## Conclusions

We show that phages with huge genomes are widespread across Earth’s ecosystems. We manually completed 35 genomes, distinguishing them from prophages, providing accurate genome lengths and complete inventories of genes, including those encoded in complex repeat regions that break automated assemblies. Even closely related phages have diversified across habitats. Host and phage migration could transfer genes relevant to medicine and agriculture (for example, genes that affect pathogenicity and antibiotic resistance) (Supplementary Information). Additional mechanisms that are relevant to medical applications involve the direct or indirect activation of immune responses. For example, some phages directly stimulate IFNγ through a TLR9-dependent pathway and exacerbate colitis^36^. Huge phages may represent a reservoir of novel nucleic acid manipulation tools with applications in genome editing and might be harnessed to improve human and animal health. For instance, huge phages equipped with CRISPR–Cas systems might be tamed and used to modulate the functions of the bacterial microbiome or eliminate unwanted bacteria.

The huge phages comprise extensive clades, suggesting that a gene inventory comparable in size to those of many symbiotic bacteria is a conserved strategy for phage survival. Overall, their genes appear to redirect the protein production capacity of the host to favour phage genes by first intercepting the earliest steps of translation and subsequently ensuring the efficient production of proteins. These inferences are aligned with findings for some eukaryotic viruses, which control every phase of protein synthesis^37^. Some phages acquired CRISPR–Cas systems with unusual compositions that may function to control host genes and eliminate competing phages.

More broadly, huge phages represent little-known biology, the platforms for which are distinct from those of small phages and partially analogous to those of symbiotic bacteria, blurring the distinctions between life and non-life. Given phylogenetic evidence for large radiations of huge phages, we wonder whether they are ancient and arose simultaneously with free-living cells, their symbionts and other phages from a pre-life (protogenote) state^38^ rather than appearing more recently through episodes of genome expansion.

## Methods

### Phage- and plasmid-genome identification

Datasets generated in the current study, those from previous research conducted by our team, the *Tara* Oceans microbiomes^42^ and the Global Oceans Virome^43^ were searched for sequence assemblies that could have derived from phages with genomes of more than 200 kb in length. Read assembly, gene prediction and initial gene annotation followed standard, previously reported methods^44–48^.

**Extended Data Fig. 6:**
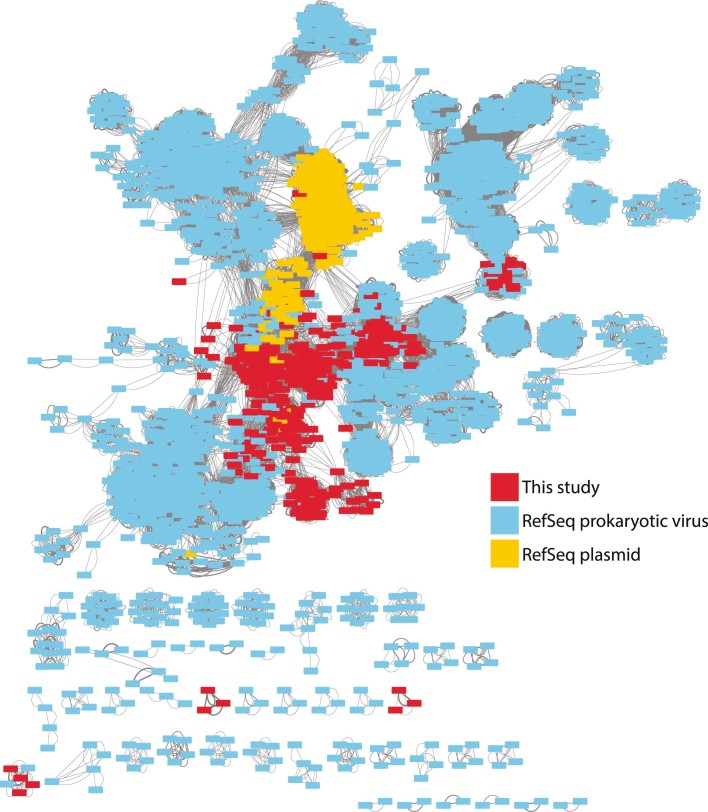
Protein-clustering network for phages and plasmids. Network analysis using vContact2 and Cytoscape^86^ based on the number of shared protein clusters between the genomes in this study, RefSeq prokaryotic virus genomes and 400 randomly sampled plasmid sequences from RefSeq. Each node represents a genome and each edge is the hypergeometric similarity (>30) between genomes based on shared protein clusters. This analysis was used to help to distinguish between the classification of genomes as phage, plasmid or unknown.

**Extended Data Fig. 7:**
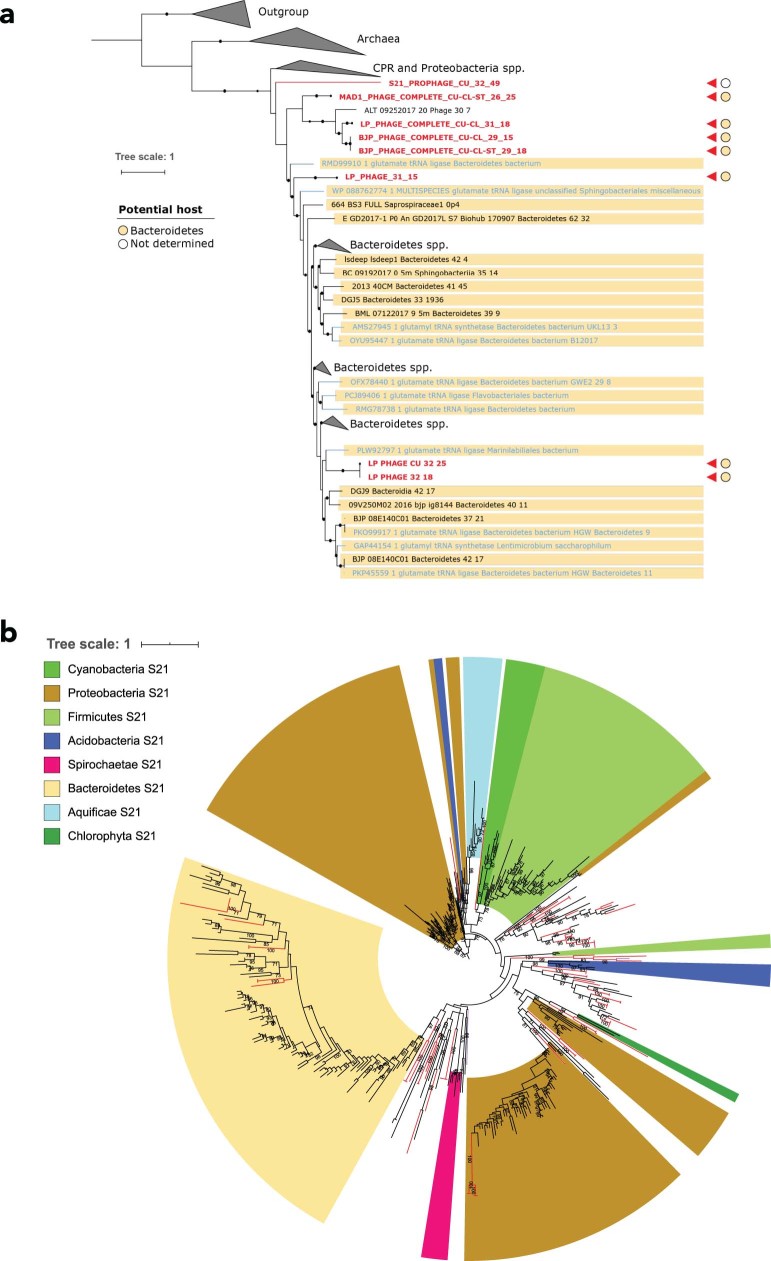
Phylogenetic analysis of tRNA synthetase. **a**, Aminoacyl tRNA synthetases were detected in many huge phages reported in this study (Supplementary Table 6). The phylogenetic subtree for glutamate-tRNA synthetase sequences from phages (red text and small triangles) that place within or close to sequences from Bacteroidetes hosts is shown as an example. Bacterial sequences from public databases are indicated by black text and those from metagenomes from which huge phage genomes were reconstructed are indicated by blue text. Coloured circles indicate the predicted phylum of the bacterial host for each phage. **b**, Phylogenetic tree of phage-encoded ribosomal protein S21 and the top RefSeq hits for each protein, constructed using IQTREE. Sequences from this study are indicated by red branches.

**Extended Data Fig. 8:**
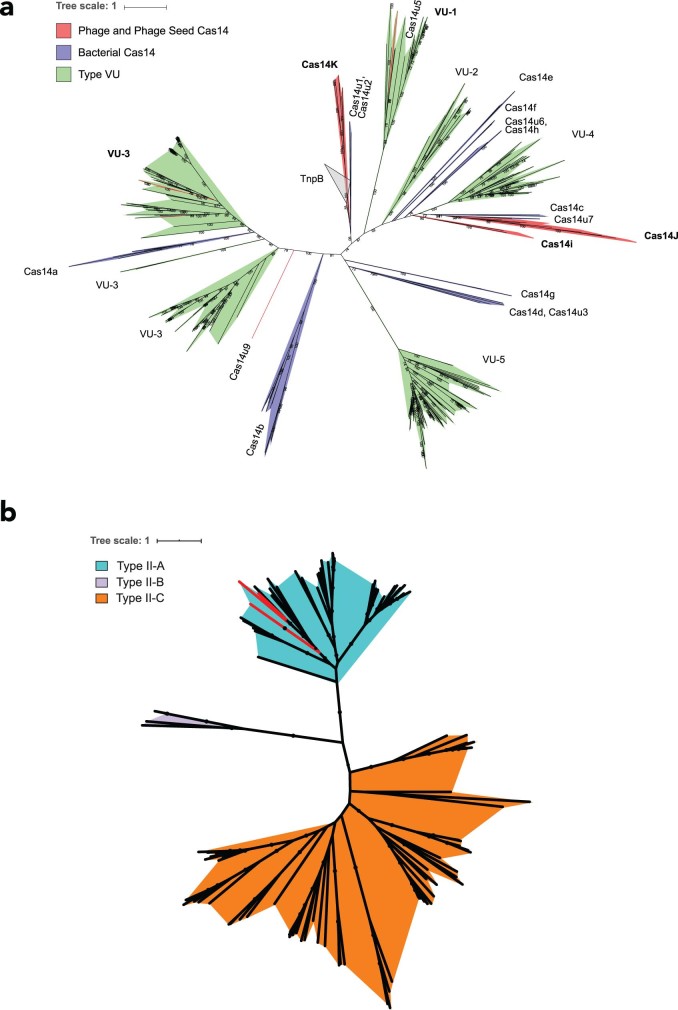
Phylogenetic trees of Cas14, CRISPR–Cas type V-U and Cas9. **a**, Phylogenetic tree for Cas14 and type V-U. **b**, Phylogenetic tree for Cas9. Sequences from this study are indicated by red branches.

**Extended Data Fig. 9:**
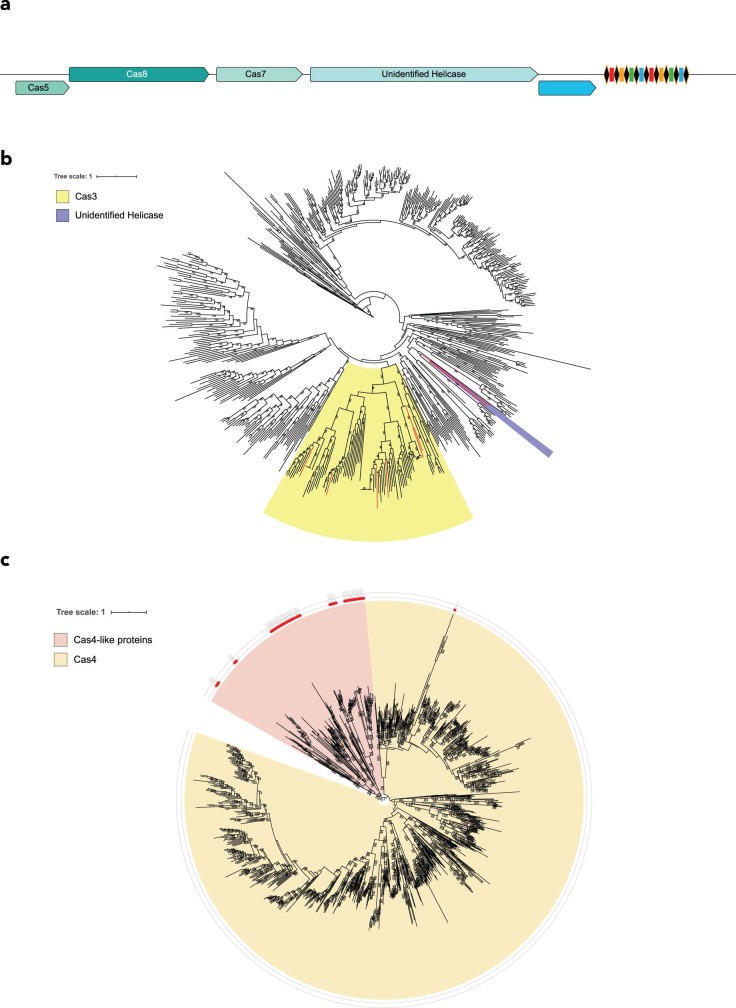
Variant type I CRISPR–Cas system and Cas4-like proteins found in the genomes of huge phages. **a**, Locus architecture for type-I variant CRISPR phages. An interesting type-I system identified in huge phages lacks Cas6 but has Cas5, which is most similar to the Cas5d protein from type I-C, in which Cas5d acts as the pre-crRNA endonuclease (a role commonly reserved for Cas6). The proposed active site residues of Cas5d are to some extent different in the Cas5 of this system, although this may still confer processing activity, as this change is also observed in other Cas6 homologues. **b**, Phylogenetic tree of superfamily 1–6 helicases, including Cas3 and the unidentified helicase in the type I-C variant system. Sequences from this study are indicated by red branches. **c**, Phylogenetic tree of Cas4, Cas4-like proteins from the phage and plasmid genomes reported here, and the top 50 RefSeq hits to the Cas4-like proteins. Cas4-like genes from this study are denoted by red circles.

**Extended Data Fig. 10:**
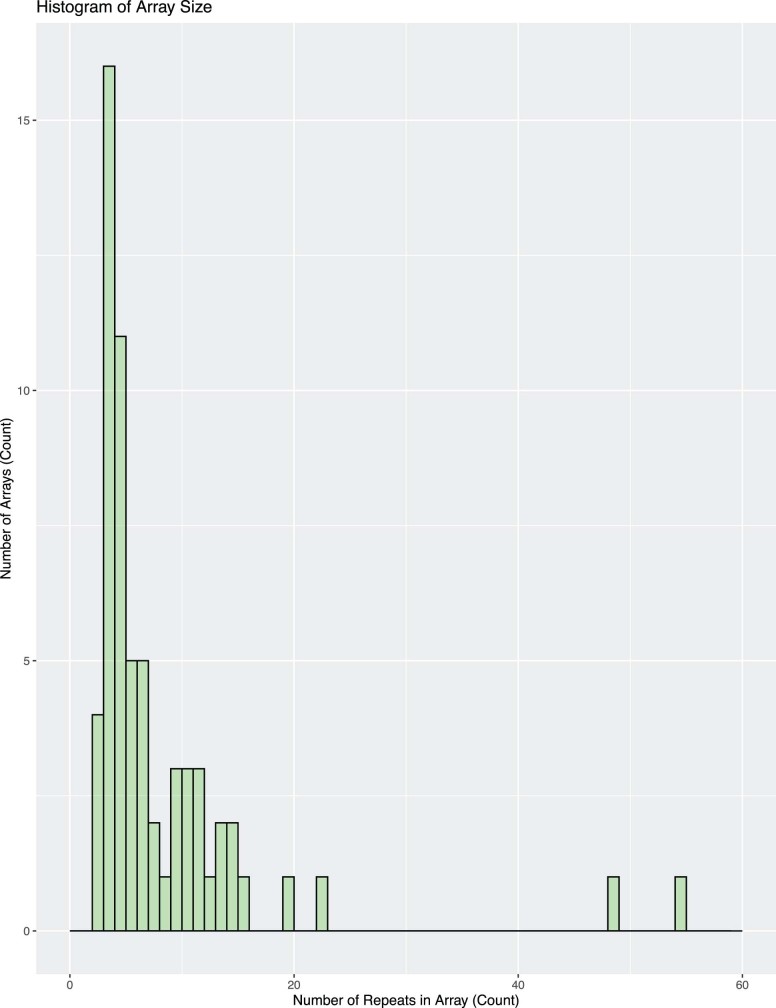
Distribution of phage- and plasmid-encoded CRISPR array sizes. The indicated count is the number of recovered repeats.

Phage candidates were initially found by retrieving sequences that were not assigned to a genome and had no clear taxonomic profile at the domain level. Taxonomic profiles were determined through a voting scheme, in which the winning taxonomy had to have more than 50% votes for each taxonomic rank on the basis of protein annotations in the UniProt and ggKbase (https://ggkbase.berkeley.edu/) databases^49^. Phages were further narrowed down by identifying sequences with a high number of hypothetical protein annotations and/or the presence of phage-specific genes, such as capsid, tail, terminase, spike, holin, portal and baseplate. All candidate phage sequences were checked throughout to distinguish putative prophages from phages. Prophages were identified on the basis of a clear transition into the host genome with a high fraction of confident functional predictions, often associated with core metabolic functions and much higher similarity to bacterial genomes. Plasmids were distinguished from phages on the basis of matches to plasmid partitioning and conjugative transfer genes. Those that did not have phage-specific genes were assigned using phylogenetic tree placement using *recA*, *polA*, *polB*, *dnaE* and the DNA sliding clamp loader gene. Phages and placement assignments were further verified using a network of protein clustering with proteins from RefSeq prokaryotic viruses and 400 randomly sampled plasmids of more than 200 kb using vContact2^50^ (Extended Data Fig. 6).

### Phage- and plasmid-genome manual curation

All classified scaffolds were tested for end overlaps indicative of circularization. Assembled sequences that could be perfectly circularized were considered potentially complete. Erroneous concatenated sequence assemblies were initially flagged by searching for direct repeats of more than 5 kb using Vmatch^51^. Potentially concatenated sequence assemblies were manually checked for multiple large repeating sequences using the dotplot and RepeatFinder features in Geneious v.9. Sequences were corrected and removed from further analysis if the corrected length was more than 200 kb.

A subset of the phage sequences was selected for manual curation, with the goal of finishing (replacing all Ns at scaffolding gaps or local misassemblies by the correct nucleotide sequences and circularization). Curation generally followed previously described methods^9^. In brief, reads from the appropriate dataset were mapped using Bowtie2 v.2.3.4.1^52^ to the de novo assembled sequences. Unplaced mate pairs of mapped reads were retained with shrinksam (https://github.com/bcthomas/shrinksam). Mappings were manually checked throughout to identify local misassemblies using Geneious v.9. N-filled gaps or misassembly corrections made use of unplaced paired reads, in some cases using reads relocated from sites to which they were mismapped. In such cases, mismappings were identified on the basis of much larger than expected paired read distances, high polymorphism densities, backwards mapping of one read pair or any combination of these. Similarly, ends were extended using unplaced or incorrectly placed paired reads until circularization could be established. In some cases, extended ends were used to recruit new scaffolds that were then added to the assembly. The accuracy of all extensions and local assembly changes were verified in a subsequent phase of read mapping. In many cases, assemblies were terminated or internally corrupted by the presence of repeated sequences. In these cases, blocks of repeated sequences as well as unique flanking sequences were identified. Reads were then manually relocated, respecting paired-read placement rules and unique flanking sequences. After gap closure, circularization and verification of accuracy throughout, end overlap was eliminated, genes were predicted and the start moved to an intergenic region, which was—in some cases—suspected to be origin on the basis of a combination of coverage trends and GC skew^53^. Finally, the sequences were checked to identify any repeated sequences that could have led to an incorrect path choice because the repeated regions were larger than the distance spanned by paired reads. This step also ruled out artefactual long phage sequences generated by end-to-end repeats of smaller phages, which occur in previously described datasets^9^.

### Structural and functional annotations

After the identification and curation of phage genomes, coding sequences and Shine–Dalgarno ribosomal binding site motifs were predicted with Prodigal using genetic code 11 (-m -g 11 -p single). The resulting coding sequences were annotated as previously described by searching UniProt, UniRef100 and KEGG^54^. Functional annotations were further assigned by searching proteins in PFAM r32^55^, TIGRFAMS r15^56^, Virus Orthologous Groups (VOG) r90 (http://vogdb.org/) and Prokaryotic Virus Orthologous Groups^57^ (pVOG). tRNAs were identified with tRNAscan-s.e. v.2.0^58^ using the bacterial model. tmRNAs were assigned using ARAGORN v.1.2.38^59^ with the genetic code of bacteria and plant chloroplasts.

Clustering of the coding sequences into families was achieved using a two-step procedure. A first protein clustering was done using the fast and sensitive protein-sequence searching software MMseqs^60^. An all-versus-all sequences search was performed using an *E*-value cut-off of 1 × 10^−3^, sensitivity of 7.5 and coverage of 0.5. A sequence similarity network was built on the basis of the pairwise similarities and the greedy set cover algorithm from MMseqs was performed to define protein subclusters. The resulting subclusters were defined as subfamilies. To test for distant homology, we grouped subfamilies into protein families using a comparison of hidden Markov models (HMMs). The proteins of each subfamily with at least two protein members were aligned using the result2msa parameter of MMseqs, and HMM profiles were built using the HHpred^61^ suite from the multiple sequence alignments. The subfamilies were then compared to each other using HHblits from the HHpred suite (with parameters -v 0 -p 50 -z 4 -Z 32000 -B 0 -b 0). For subfamilies with probability scores of at least 95% and coverage at least 0.50, a similarity score (probability × coverage) was used as weight of the input network in the final clustering using the Markov clustering algorithm^62^, with 2.0 as the inflation parameter. These clusters were defined as the protein families. Protein sequences were functionally annotated on the basis of their best hmmsearch match (v.3.1) (*E*-value cut-off 1 × 10^−3^) against an HMM database constructed on the basis of orthologous groups defined by the KEGG database^63^ (downloaded on 10 June 2015). Domains were predicted using the same hmmsearch procedure against the PFAM r31 database^55^. The domain architecture of each protein sequence was predicted using the DAMA software^64^ (default parameters). SIGNALP^65^ (v.4.1) (parameters, -f short -t gram+) and PSORT^66^ v.3 (parameters, --long --positive) were used to predict the putative cellular localization of the proteins. Prediction of transmembrane helices in proteins was performed using TMHMM^67^ (v.2.0) (default parameters). Hairpins (palindromes, based on identical overlapping repeats in the forward and reverse directions) were identified using the Geneious Repeat Finder and located across the dataset using Vmatch^51^. Repeats of more than 25 bp with 100% similarity were tabulated.

### Reference genomes for size comparisons

RefSeq r92 genomes were recovered using the NCBI Virus portal and selecting only complete dsDNA genomes with bacterial hosts. Genomes from a previously published study^14^ were downloaded from IMG/VR and only sequence assemblies that were labelled ‘circular’ with predicted bacterial hosts were retained. Given the presence of sequences in IMG/VR that were based on erroneous concatenations, we only considered sequences from this source that were more than 200 kb; however, a subset of these was removed as artefactual sequences.

### Alternative genetic codes

In cases in which the gene prediction using the standard bacterial code (code 11) resulted in seemingly anomalously low coding densities, potential alternative genetic codes were investigated. In addition to making a prediction using the fast and accurate genetic code inference and logo^68^ (FACIL) web server, we identified genes with well-defined functions (for example, polymerase or nuclease) and determined the stop codons terminating genes that were shorter than expected. We then repredicted genes using GLIMMER3 v.1.5^69^ and Prodigal with TAG not interpreted as a stop codon. Other combinations of repurposed stop codons were evaluated and candidate codes (for example, code 6, with only one stop codon) were ruled out owing to unlikely gene-fusion predictions.

### Large terminase subunit and MCP phylogenetic analyses

The phylogenetic tree of the large terminase subunit was constructed by recovering large terminases from the aforementioned protein-clustering and annotation pipeline. The coding sequences that matched with >30 bitscore to PFAM, TIGRFAMS, VOG and pVOG were retained. Any coding sequence that had a hit to large terminase, regardless of bitscore, was searched using HHblits^70^ against the uniclust30_2018_08 database. The resulting alignment was then further searched against the PDB70 database. Remaining coding sequences that clustered in protein families with a large terminase HMM were also included after manual verification. Detected large terminases were manually verified using the HHPred^70^ and jPred^71^ webservers. Large terminases from the >200-kb phage genomes^14^ and all >200-kb complete dsDNA phage genomes from RefSeq r92 were also included by protein family clustering with the phage-coding sequences from this study. The resulting terminases were clustered at 95% amino acid identity to reduce redundancy using CD-HIT^72^. Smaller phage genomes were included by searching the resulting coding sequences set against the full RefSeq protein database and retaining the top 10 best hits. Those hits that had no large terminase match against PFAM, TIGRFAMS, VOG or pVOG were removed from further consideration and the remaining set was clustered at 90% amino acid identity. The final set of large terminase coding sequences that were more than 100 amino acids in length were aligned using MAFFT^73^ v.7.407 (--localpair --maxiterate 1000) and poorly aligned sequences were removed and the resulting set was realigned. The phylogenetic tree was inferred using IQTREE v.1.6.6 using automatic model selection^74^. The phylogenetic tree of MCP genes was constructed by retrieving all MCPs annotated by combining the PFAM annotations of protein families and direct annotations by PFAM, TIGRFAMS, VOG and pVOG. Reference MCP gene sequences were collected using the same strategy and sources as for the large terminase subunit tree. The resulting set was further screened by searching against PFAM, TIGRFAMS, VOG and pVOG and removing matches that had no large terminase match regardless of bitscore. The final set of MCP sequences were aligned with MAFFT(--localpair --maxiterate 1000) and the phylogenetic tree was constructed using IQTREE with automatic model selection and 1,000 bootstrap replicates.

### Whole-genome scale clustering

To identify phage genomes that were closely related at the whole-genome level, we compared sequences using whole-genome alignments. The goal of this analysis was to further corroborate the identified phylogenetic clades and test for the presence of very similar phages in different habitats and environments. Genomes grouped together in the primary clusters from dRep v.2^75^ were evaluated for genome alignment using Mauve^76^ within Geneious v.9.

### CRISPR–Cas locus and target detection

Phage- and host-encoded CRISPR loci (repeats and spacers) were identified using a combination of MinCED (https://github.com/ctSkennerton/minced) and CRISPRDetect^77^. A custom database of Cas genes was built by collecting Cas gene sequences from previous studies^23,25,78–82^ and built with MAFFT (--localpair --maxiterate 1000) and hmmbuild. The coding sequences from this study were searched against the HMM database using hmmsearch with *E* < 1 × 10^−5^. Matches were checked using a combination of hmmscan and BLAST searches against the NCBI nr database and manually verified by identifying colocated CRISPR arrays and Cas genes. Spacers extracted from between repeats of the CRISPR locus were compared to sequences assemblies from the same site using BLASTN-short^83^. Matches with alignment length >24 bp and ≤1 mismatch were retained and targets were classified as bacteria, phage or other. CRISPR arrays that had ≤1 mismatch, were further searched for more spacer matches in the target sequence by finding more hits with ≤3 mismatches.

### Host identification

The phylum affiliations of bacterial hosts for phage and plasmid-like sequences were predicted by considering the UniProt taxonomic profiles of every coding sequence for each phage genome. The phylum level matches for each phage genome were summed and the phylum with the most hits was considered as the potential host phylum. However, only cases in which this phylum that had 3× as many counts as the next most-counted phylum were assigned as the tentative phage host phylum. Phage hosts were further assigned and verified using the CRISPR-targeting strategy describe above with the phage and plasmid-like genomes as targets. CRISPR arrays were predicted on all sequence assemblies from the same site that each phage genome was reconstructed. Sequence assemblies containing spacers with a match of length >24 bp and ≤1 mismatch were used to infer phage–host relationships. In all cases, the predicted host phylum based on taxonomic profiling and CRISPR targeting were in complete agreement. Similarly, the phyla of hosts were predicted on the basis of phylogenetic analysis of phage genes also found in host genomes (for example, involved in translation and nucleotide reactions). Inferences based on computed taxonomic profiles and phylogenetic trees were also in complete agreement.

### Phage-encoded tRNA synthetase trees

Phylogenetic trees were constructed for phage-encoded tRNA synthetase, ribosomal and initiation factor protein sequences using a set of the closest reference sequences from NCBI and bacterial genomes from the current study. The tRNA synthetases were identified on the basis of annotation of genes using the standard ggKbase pipeline (see above), and confirmed by HMMs with datasets from TIGRFAMs. For each type of tRNA synthetase, references were selected by comparing all of the corresponding genes of this type against the NCBI nr database using DIAMOND v.0.9.24^84^, their top 100 hits were clustered by CD-HIT using a 90% similarity threshold^72^. The phylogenetic tree of each tRNA synthetase was constructed using RAxML v.8.0.26^85^ with the PROTGAMMALG model.

### Reporting summary

Further information on research design is available in the Nature Research Reporting Summary linked to this paper.

## Online content

Any methods, additional references, Nature Research reporting summaries, source data, extended data, supplementary information, acknowledgements, peer review information; details of author contributions and competing interests; and statements of data and code availability are available at 10.1038/s41586-020-2007-4.

## Supplementary information

Supplementary Discussion Supplementary Discussion: Additional discussion in support of study findings.

Reporting Summary

Supplementary Methods This file contains the publications that were used as sequence data sources.

Supplementary Tables This file contains Supplementary Tables 1-10 with a guide.

Supplementary Data Supplementary Data: Genbank files of all phage, plasmid, and unknown sequences in this study; major capsid, terminase, tRNA synthetase, Cas9, rpS21, 1-6 helicases, and Cas4-like phylogenetic trees; dendrogram of protein clustering; alignments of Cas gene catalytic residues; protein sequences of new Cas12 and Cas14 subtypes; bacterial sequences that target phage, plasmid, and unknown sequences using CRISPR.

Supplementary Data Genbank files for all genome.

Supplementary Data tRNA synthetase trees (Newick files).

Supplementary Data Alignment file showing conservation of catalytic residues implicated in DNase activity in Cas12J, Cas14 and Type VU, Cas9, Cas3, Cas10.

Supplementary Data Sequences of bacterial scaffolds that target phage or plasmids reported in this study and sequences of scaffold targets of phage/plasmid encoded CRISPR protospacers.

Supplementary Data Proteins sequences for new Cas12 and Cas14 subtypes.

Supplementary Data Full terminase and capsid protein phylogenetic trees (Newick files).

Supplementary Data Dendrogram of presence/absence of protein families across phage genomes.

## Data Availability

GenBank files for all genomes are provided as Supplementary Information. Sequence reads and genomes have been deposited at the European Nucleotide Archive (ENA) under project PRJEB35371. Genomes have been deposited at ENA under accessions ERS4026114–ERS4026474. Reads are available at ENA under accessions ERS4025670–ERS4025731. Read accessions and genome accessions for each phage genome are included in Supplementary Table 1.

## Code availability

The custom code used to analyse the genomes is available at http://www.github.com/rohansachdeva/assembly_repeats.
