# Supplementary material for: Clades of huge phages from across Earth’s ecosystems
Source: Nature. 2020 Feb 12;578(7795):425–31. doi: 10.1038/s41586-020-2007-4 (PMC7162821; doi:10.1038/s41586-020-2007-4)
Supplement: Supplementary file 1 — Supplementary Discussion: Additional discussion in support of study findings. [file 41586_2020_2007_MOESM1_ESM.pdf]

nature

https://doi.org/10.1038/s41586-020-2007-4

Supplementary information

Clades of huge phages from across Earth's ecosystems

In the format provided by the authors and unedited

Basem Al-Shayeb, Rohan Sachdeva, Lin-Xing Chen, Fred Ward, Patrick Munk, Audra Devoto, Cindy J. Castelle, Matthew R. Olm, Keith Bouma-Gregson, Yuki Amano, Christine He, Raphaël Méheust, Brandon Brooks, Alex Thomas, Adi Lavy, Paula Matheus-Carnevali, Christine Sun, Daniela S. A. Goltsman, Mikayla A. Borton, Allison Sharrar, Alexander L. Jaffe, Tara C. Nelson, Rose Kantor, Ray Keren, Katherine R. Lane, Ibrahim F. Farag, Shufei Lei, Kari Finstad, Ronald Amundson, Karthik Anantharaman, Jinglie Zhou, Alexander J. Probst, Mary E. Power, Susannah G. Tringe, Wen-Jun Li, Kelly Wrighton, Sue Harrison, Michael Morowitz, David A. Relman, Jennifer A. Doudna, Anne-Catherine Lehours, Lesley Warren, Jamie H. D. Cate, Joanne M. Santini & Jillian F. Banfield✉

Clades of huge phage from across Earth's ecosystems

Basem Al-Shayeb, Rohan Sachdeva et al.

Supplementary Discussion

Phage with low coding densities suggestive of use of an alternative genetic code

We examined phage genomes whose coding density was very low when genes were predicted with bacterial code 11 (list below). For the cases with the lowest coding densities we considered predictions in which each of the normal stop codons encoded an amino acid ('recoded') and cases where two of three stop codons were recoded. We also used FACIL[1] to predict the genetic code and found that for many genomes, multiple changes in coding (not all involving stop codons) were suggested, although in many cases tentatively. It seems likely that FACIL may not be ideally suited to resolving phage coding, given that it was trained on sequences from bacteria, archaea, and organellar genomes.

In general, examination of sequences suggested that the most plausible choice (due to increased coding density and lack of extremely large open reading frames) involved TAG coding for an amino acid. Because the phage proteins studied here are so divergent from proteins in public databases, it was not possible to evaluate the credibility of the vast majority of predicted proteins to evaluate alternative code predictions. Thus, we first re-predicted and functionally re-annotated the genomes with the lowest predicted code 11 coding % using code 16 as a proxy for TAG repurposing and evaluated cases where genes with clear annotations (*e.g.*, polymerase, *gyrA*) were split with one code but not another. For genomes of close to or >80% coding density, code 11 and code 16 (and some other choices) predicted open reading frames differed relatively little. In most cases, the presence of a suppressor tRNA with the expected anticodon CTA supports repurposing of the TAG stop codon.

Given the possibility that code changes other than repurposing of stop codons may have occurred (as suggested by FACIL) and as we are unable to confidently determine what stop codons may

code for, we chose not to repredict and reannotate the phage genomes. In most cases, comparisons of the code choices indicate that reprediction would not result in major changes in the predicted proteomes.

Alternative coding was previously reported by Ivanova *et al.*[2] and Devoto *et al.*[3] Interestingly, all but one of the phage with the lowest coding densities predicted using the bacterial genetic code 11 derives from a human or animal microbiome, possibly reflecting the Firmicute host association (see **Extended Data Figure 2**). Ivanova *et al.* suggest that “bacteriophages can infect hosts with a different genetic code and demonstrate phage-host antagonism based on code differences”. In a survey of metagenome data, these authors predicted one phage with stop codon reassignment to TAG reassigned to Ser and 14 to Gln. **Extended Data Figure 4** provides examples of gene predictions using the standard bacterial and alternative code.

**Genome Name**	**code_11 coding %**	**Phylum of predicted host**	**Notes**
M05_PHAGE_COMPLETE_32_31	63.62	Firmicutes	TAG likely recoded based on many genes, e.g., DNA polymerase, DNA specific exonuclease. Suppressor tRNA with anticodon CTA is predicted. Ile tRNA predicted to have an intron.
F27_PHAGE_COMPLETE_34_39	65.73	Unknown	Chlamydiae profile error tracked back to Sanger Inst. metagenome misclassification; TAG likely recoded. No Sup tRNA predicted
AOT-PERU_PHAGE_COMPLETE_CU-CL-ST_331_34	70.59	Unknown	TAG recoded based on split gyrA with well conserved sequences. Suppressor tRNA with anticodon

			CTA is predicted. A Glu tRN predicted to have an intron.
F09_PHAGE_COMPLETE_33_145	73.13	Firmicutes	Undetermined. Suppressor tRNA with anticodon CTA is predicted.
LP_PHAGE_22_14	74.25	Unknown	TAG recoded based on split thymidylate and dCTP pyrophosphatase synthase with well conserved sequences. Suppressor tRNA with anticodon CTA is predicted.
M01_PHAGE_COMPLETE_33_48	75.42	Firmicutes	TAG recoded based on an conserved integrase interrupted by two stop codons. Suppressor tRNA with anticodon CTA is predicted. tRNA Gly, Gln, Ile are predicted to have introns.
A6_PHAGE_COMPLETE_CU-CL_34_1	76.61	Firmicutes	TAG recoded based on interruption of conserved terminase, portal, 2 polymerases, primase. Suppressor tRNA with anticodon CTA is predicted.
F21_PHAGE_COMPLETE_34_7	76.7	Firmicutes	TAG recoded based on interruption of conserved integrase. Suppressor tRNA with anticodon CTA is predicted. Introns predicted in tRNA Gln, Pro, Glu, Ile.
H18-	77.59	Firmicutes	TAG recoded based on interruption

TANZANIA_PHAGE_33_33			of conserved ribonucleoside reductase. Suppressor tRNA with anticodon CTA is predicted. Introns predicted in tRNA Glu, Ile, Gly.
SALIVA_PHAGE_50_20	80.23	Unknown	TAG recoding not supported as ORFs almost the same as for code 11. No Sup tRNA predicted.

Palindromic hairpins in phage genomes

About half of the phage genomes have one to fifty sequences >25 bp in length that fold into perfect hairpins (**Table S9**). The palindromes (sequences with dyad symmetry) are almost exclusively intergenic and each is unique within a genome. Some, but not all, are predicted to be Rho-independent terminators, thus provide clues regarding genes that function as independently regulated units (Methods). However, some palindromes are larger: up to 74 bp in length, with 34 genomes having examples ≥40 nt in length. These may have alternative or additional functions, such as modulating translation initiation and processivity.

Phage-specific proteins consistent with classification of sequences as phage

Phage proteins identified in the genomes include Terminase, Baseplate_J, bacteriophage J protein, GP11 baseplate wedge protein, GP38 Phage tail fiber adhesin, Gp45_2, GP88 of unknown function, GPW_gp25 baseplate protein, Phage_cap_E phage major capsid protein E involved with the stabilization of the condensed form of the DNA molecule in phage heads, Phage_fiber_2 phage tail fiber repeat, Phage_gp49_66 functionally uncharacterized, Phage_gp53 base plate wedge protein 53, Phage-Gp8 viral baseplate structural protein, Phage_head_chap head assembly gene product Phage_head_fibr Head fiber protein, Phage_min_tail / PhageMin_Tail**,** Phage_sheath_1 phage tail sheath proteins**,** Phage_sheath_1C**,** Phage_T4_gp36 phage T4 tail fiber, Phage-tail_3 putative phage tail protein, Phage_tail_APC phage tail assembly chaperone protein**,** Phage_tail_L phage minor tail protein L**,** Phage_tail_NK Sf6-type phage tail

needle knob or tip of some Caudovirales**,** Prophage_tail prophage endopeptidase tail**,** Sipho_tail**,** T4-gp15_tss tail-sheath-stabiliser or tail-terminator protein of T4-like myoviridae phage, T4_gp9_10 family components of the viral baseplate, Gp9 (P10927) connects the long tail fibers of the virus to the baseplate and triggers tail contraction after viral attachment to a host cell, T4_tail_cap for the assembly and length regulation of the tail tube of bacteriophage T4, Tail_P2_I tail proteins. A full summary of all genes is provided in
Table S4.

Analysis of similar huge phage genomes across ecosystems and cohorts

Clades of phage from more than one ecosystem type or cohort (in the case of humans) that are sufficiently closely related that whole genome alignments (or major parts of them) could be established using Mauve. Twenty examples were identified, two of which include phage sampled from three different locations/habitat types. These phage also group together in the terminase trees (where terminases were identified) or by protein clustering analysis. Not listed are the many groups of closely related phage that were found in a single habitat type

Phage tRNAs with introns

A few tRNA genes in some genomes have introns of unclassified types, similar to results reported for Lak phage by Devoto *et al*. For example, for A3_PHAGE_COMPLETE_CU-CL_34_1, there are three tRNAs predicted to contain introns (see Methods). Also see summary information for phage with low coding densities, above.

Phylogenetic analysis of tRNA synthetase genes

We identified aminoacyl tRNA synthetase genes on phage genomes, up to 15 per genome (BJP_PHAGE_COMPLETE_CU-CL-ST_29_18). Also identified were genes involved in tRNA modification, such as lysidine synthetase tRNA(Ile). For full listing, see **Table S6**, and for more information, see **Table S4**. The full phylogenetic trees for tRNA synthetases are provided as Supplemental Data. In general, phage tRNA synthetases place within groups of sequences from

bacteria, typically those related to the presumed phage host. An example is shown in **Extended Data Figure 7A**.

Other phage genes of potential importance

1. Augment host metabolism: Phage genes predicted to augment host metabolism include photosynthesis genes in phage that infect both cyanobacteria and proteobacteria, sulfur metabolism, biosynthesis of cofactors, central carbon metabolism, cell surface proteins, cell defense. For more information, see **Tables S4A, B**). To our knowledge, we also report the first dinitrogenase reductase ADP-ribosyltransferases (DRAT) in phages. DRATs can control the input of bioavailable nitrogen by inhibiting nitrogenase genes, and thus stopping nitrogen fixation.

2. Defense: Phage_Mu_Gam, which protects linear double stranded DNA from exonuclease degradation in vitro and in vivo [4], Phage_pRha, possibly a phage regulatory protein that interferes with infection of bacterial strains that lack integration host factor (IHF). Also identified were genes related to sporulation, heat shock response. Interestingly, perhaps, F21_PHAGE_COMPLETE_34_7 and A9 encode a eukaryotic type RNA directed RNA polymerase (RdRP) that might function in a way analogous to RNAi. Eukaryotic RdRP can act as post-transcriptional gene silencer by creating dsRNA from mRNA templates[5]. We also discovered two (possibly redundant) genomes from previously published jumbophages that have eukaryotic-like RdRP: EF583821.1[6] and NC_009760.1[7]. Ten genomes are predicted to have RdRp.

3. Numerous transposases; by far the most common are fam00026 belonging to the IS605 superfamily, which is speculated to be part of the ancestral origin of Type V CRISPR-Cas systems: HTH_OrfB_IS605 (PF12323) + OrfB_IS605 (PF01385) + OrfB_Zn_ribbon (PF07282).

4. Antimicrobial and lipopolysaccharides: Phage A3_PHAGE_COMPLETE_CU-CL_34_1 has three closely placed sequential enzymes of the streptomycin biosynthesis pathway and another gene in this pathway, so it can convert D-glucose-1P to dTDP-L-rhamnose, which is an

immediate precursor of rhamnose moiety in the O antigen of lipopolysaccharide in gram-negative bacteria. For more information, see **Table S4**.

Phage-encoded phage shock proteins: These are not typically reported in phage, to our knowledge. However, they are widespread in huge phage predicted to infect Actinobacteria, Bacteroidetes, Firmicutes, Planctomycetes and Proteobacteria. Proteins include PspA, PspC, PspE. PspA, for example, has been linked to virulence (see **Table S4**).

Phage-encoded phage receptors: We identified some phage encoded proteins that may serve as phage receptors, an intriguing hint that such proteins are produced by the host and exported to the cell surface where they enable entry of new phage. For more information, see **Table S4**.

Phage targeting of translation-relevant genes

We observed a case where a phage spacer in M05_PHAGE_COMPLETE_47_87 is identical to a protospacer within the sigma 70 transcription factor encoded in its suspected Firmicute host genome, and the phage carries its own sigma 70 transcription factor within its genome.

We also noted one case (RIF_PHAGE_CU_29_17) where a phage CRISPR spacer targets the host Class II tRNA synthetase, likely a Glycyl tRNA synthetase. This phage carries its own glycyl tRNA. Although we did not identify a phage-type Glycyl tRNA synthetase, the genome encodes a Class II tRNA synthetase of unresolved specificity that may have this function. If so, silencing of the bacterial tRNA synthetase may enable the phage to substitute its own translational machinery for that of its host.

Lack of CRISPR targeting of phage with CRISPR systems

The lack of cases where phage with CRISPR systems are targeted by a bacterial CRISPR system may be coincidence, or the results might indicate that it is disadvantageous for a bacterial host to carry a CRISPR system targeting CRISPR phage. A phage encoding a CRISPR-Cas system may be selected against if the host has a spacer targeting the phage and a compatible CRISPR system (*e.g.*, with a common array), as the host could use the phage's own Cas proteins against it. This would result in the lack of identification of a host-targeted CRISPR phage within the sample.

Alternatively, phage may have a mechanism that prevents the host from acquiring a spacer targeting it.

Phage CRISPR-Cas systems

Observations of phage targeting other phage parallel prior reports of a phage-associated locus that targets suspected prophage[8] and of a locus on a plasmid-like phage that targets a phage from the same system[9]. Many of the phage lack complete CRISPR-Cas systems, or harbor a solo array with no Cas protein within the gene neighborhood. The phage may be providing an array as a guiding system for the host to use against its competing phage, or the host Cas proteins may substitute for those missing in the CRISPR phage (including acquisition systems) in the situations where the host system is compatible, or the surveillance complex may be formed and bound to the target protospacers, inhibiting transcription. Additionally, the array may hybridize to its target and inhibit transcription in an RNAi-like mechanism. If the repeats in the CRISPR phage are compatible with those of the host, they may also acquire spacers via homologous recombination, or use homologous recombination to integrate into their host genome.

Plasmid CRISPR-Cas systems

Plasmid-encoded CRISPR spacers target genes within predicted hosts, other plasmid-like sequences, and in one case, a Tn7-like transposon within the predicted host.

Phage genome-compartments, or "nuclei" possibly widespread in huge phage

Also, identified were proteins from a family that includes the three previously described pseudomonas homologs forming the nucleic proteinaceous barrier (gp105 in 201Φ2-1, gp54 in ΦKZ, gp53 in ΦPA3) (Chaikeeratisak et al. 2017).

References

1. Dutilh, B. E. *et al.* FACIL: Fast and Accurate Genetic Code Inference and Logo. *Bioinformatics* **27**, 1929–1933 (2011).
2. Ivanova, N. N. *et al.* Stop codon reassignments in the wild. *Science* **344**, 909–913 (2014).
3. Devoto, A. E. *et al.* Megaphages infect Prevotella and variants are widespread in gut microbiomes. *Nat Microbiol* (2019). doi:10.1038/s41564-018-0338-9
4. Bhattacharyya, S. *et al.* Phage Mu Gam protein promotes NHEJ in concert with Escherichia coli ligase. *Proc. Natl. Acad. Sci. U. S. A.* **115**, E11614–E11622 (2018).
5. Sijen, T. *et al.* On the role of RNA amplification in dsRNA-triggered gene silencing. *Cell* **107**, 465–476 (2001).
6. Hardies, S. C., Thomas, J. A. & Serwer, P. Comparative genomics of Bacillus thuringiensis phage 0305φ8-36: defining patterns of descent in a novel ancient phage lineage. *Virol. J.* **4**, 97 (2007).
7. Thomas, J. A. *et al.* Complete genomic sequence and mass spectrometric analysis of highly diverse, atypical Bacillus thuringiensis phage 0305ϕ8–36. *Virology* **368**, 405–421 (2007).
8. Sebaihia, M. *et al.* The multidrug-resistant human pathogen Clostridium difficile has a highly mobile, mosaic genome. *Nat. Genet.* **38**, 779–786 (2006).
9. Minot, S. *et al.* The human gut virome: inter-individual variation and dynamic response to diet. *Genome Res.* **21**, 1616–1625 (2011).
