# Supplementary material for: Clades of huge phages from across Earth’s ecosystems
Source: Nature. 2020 Feb 12;578(7795):425–31. doi: 10.1038/s41586-020-2007-4 (PMC7162821; doi:10.1038/s41586-020-2007-4)
Supplement: Supplementary file 2 — Reporting Summary [file 41586_2020_2007_MOESM2_ESM.pdf]

nature research

Corresponding author(s): Jillian F Banfield

Last updated by author(s): Nov 13, 2019

Reporting Summary

Nature Research wishes to improve the reproducibility of the work that we publish. This form provides structure for consistency and transparency in reporting. For further information on Nature Research policies, see Authors & Referees and the Editorial Policy Checklist.

Statistics

For all statistical analyses, confirm that the following items are present in the figure legend, table legend, main text, or Methods section.

n/a	Confirmed	
☐	☒	The exact sample size (n) for each experimental group/condition, given as a discrete number and unit of measurement
☒	☐	A statement on whether measurements were taken from distinct samples or whether the same sample was measured repeatedly
☐	☒	The statistical test(s) used AND whether they are one- or two-sided<br>*Only common tests should be described solely by name; describe more complex techniques in the Methods section.*
☒	☐	A description of all covariates tested
☒	☐	A description of any assumptions or corrections, such as tests of normality and adjustment for multiple comparisons
☐	☒	A full description of the statistical parameters including central tendency (e.g. means) or other basic estimates (e.g. regression coefficient) AND variation (e.g. standard deviation) or associated estimates of uncertainty (e.g. confidence intervals)
☐	☒	For null hypothesis testing, the test statistic (e.g. F, t, r) with confidence intervals, effect sizes, degrees of freedom and P value noted<br>*Give P values as exact values whenever suitable.*
☒	☐	For Bayesian analysis, information on the choice of priors and Markov chain Monte Carlo settings
☒	☐	For hierarchical and complex designs, identification of the appropriate level for tests and full reporting of outcomes
☐	☒	Estimates of effect sizes (e.g. Cohen's d, Pearson's r), indicating how they were calculated

Our web collection on statistics for biologists contains articles on many of the points above.

Software and code

Policy information about availability of computer code

Data collection

Geneious v9.1.8 (Licensed, paid version used in this study, free versions available)
BBmap v37.5
IDBA_UD v1.1.1
Bowtie2 v2.3.4.1
MEGAHIT v1.1.3
SPAdes v3.11.1

Data analysis

vContact2
Vmatch v2.3
Prodigal v2.6.3
tRNAscan-SE v2.0
ARAGORN v1.2.38
MMseqs2 Version: 9f493f538d28b1412a2d124614e9d6ee27a55f45
HHsuite v3.0.3
CD-HIT v4.6.8
SignalP v4.1
DAMA v1.0
PSORT v3.0
TMHMM v2.0
MAFFT v7.407
DIAMOND v0.9.24
RAxML v8.0.26
IQTREE v1.6.6
HMMER v3.1b2
GLIMMER3 v1.5

MinCED v0.2.0
https://github.com/rohansachdeva/assembly_repeats v0

For manuscripts utilizing custom algorithms or software that are central to the research but not yet described in published literature, software must be made available to editors/reviewers. We strongly encourage code deposition in a community repository (e.g. GitHub). See the Nature Research guidelines for submitting code & software for further information.

Data

Policy information about availability of data

All manuscripts must include a data availability statement. This statement should provide the following information, where applicable:

- Accession codes, unique identifiers, or web links for publicly available datasets
- A list of figures that have associated raw data
- A description of any restrictions on data availability

Accession numbers for each genome and reads are provided in the data availability statement and in Table S1. Genbank files for each genome are also provided as supplementary data.

Field-specific reporting

Please select the one below that is the best fit for your research. If you are not sure, read the appropriate sections before making your selection.

☒ Life sciences ☐ Behavioural & social sciences ☐ Ecological, evolutionary & environmental sciences

For a reference copy of the document with all sections, see nature.com/documents/nr-reporting-summary-flat.pdf

Life sciences study design

All studies must disclose on these points even when the disclosure is negative.

Sample size	The sample size was chosen to provide a high breadth of ecosystem coverage for the recovery of huge phage genomes. Accordingly, there were no statistical methods to determine sample sizes. Data were compiled from multiple sources where virus-like genomes could be found. Data were compiled from multiple sources wherever virus-like genomes could be found. Samples from each sampling site is listed in Table 1 and Table S1
Data exclusions	IMG/VR phage data was excluded because of the prevalence of artifactual concatenated viral sequence assemblies. This was pre-established based on criteria in Devoto et al. 2019.
Replication	Near identical phage genomes were recovered from multiple independent samples, verifying sequence assembly and genome reconstruction. Host identification was verified by a combination of CRISPR targeting, phylogenetic analysis of ribosomal proteins, and phylum-level taxonomic profiles. Annotations were verified across multiple databases.
Randomization	Randomization is not applicable to this because the reported study is a survey of huge phage genomes across many ecosystems and not dependent on experimental outcomes.
Blinding	Blinding was not performed because it was not applicable to this study. This study was a survey of huge phage genomes across global populations, and was not dependent on trial outcomes

Reporting for specific materials, systems and methods

We require information from authors about some types of materials, experimental systems and methods used in many studies. Here, indicate whether each material, system or method listed is relevant to your study. If you are not sure if a list item applies to your research, read the appropriate section before selecting a response.

Materials & experimental systems

n/a	Involved in the study
☒	☐ Antibodies
☒	☐ Eukaryotic cell lines
☒	☐ Palaeontology
☒	☐ Animals and other organisms
☒	☐ Human research participants
☒	☐ Clinical data

Methods

n/a	Involved in the study
☒	☐ ChIP-seq
☒	☐ Flow cytometry
☒	☐ MRI-based neuroimaging
