# Supplementary material for: Clades of huge phages from across Earth’s ecosystems
Source: Nature. 2020 Feb 12;578(7795):425–31. doi: 10.1038/s41586-020-2007-4 (PMC7162821; doi:10.1038/s41586-020-2007-4)
Supplement: Supplementary file 3 — This file contains the publications that were used as sequence data sources. [file 41586_2020_2007_MOESM3_ESM.pdf]

Clades of huge phage from across Earth's ecosystems

Basem Al-Shayeb, Rohan Sachdeva et al.

Supplementary Methods

Data sources

The following publications were used as sequence data sources (Table S1 has detailed information on the source of each phage genome):

Anantharman et al. 2016[1], Borton et al. 2018[2], Bouma-Gregson et al. 2019[3], Brown et al. 2015[4], Dalcin Martins et al. 2018[5], David et al. 2015[6], Devoto et al. 2019[7], Finstad et al. 2017[8], Goltsman et al. 2018[9], Hug et al. 2015[10], Hug et al. 2016[11], Kantor et al. 2017[12], Karsenti et al. 2011[13], Lavy et al. 2019[14], Obregon-Tito et al. 2015[15], Olm and West et al. 2019[16], Probst et al. 2018[17], Rahman et al. 2017[18], Rampelli et al. 2015[19], Roux et al. 2016[20], Sharrar et al. 2019[21], Sieber et al. 2018[22], Solden et al. 2018[23], Tung et al. 2015[24], Youssef et al. 2019[25]

Analysis of replication style and location of the origin of replication

We calculated the GC skew (G-C)/(G+C) and cumulative GC skew[26] across the phage chromosome sequences to predict the style of replication (bidirectional vs. unidirectional) and the location of the origin of replication (in cases where replication proceeds bidirectionally from the origin to terminus). For curated, complete genomes, 39% display a pattern consistent with bidirectional replication (although the start may not be placed in this region), 36% consistent with unidirectional replication. For the remainder (circularized and not circularized), 29% display a pattern consistent with bidirectional and 29% consistent with unidirectional replication. The remaining cases could not be clearly classified, most often due to very low overall GC skew. For examples see **Extended Data Figure 2.**

Artifactual assemblies in public databases

In addition to examples reported by Devoto *et al.*[7] from the IMG-VR database, we uncovered the following sequences in IMG-VR or IMG that are comprised of artifactual perfect concatenations of smaller sequences:

3300000060_____PaMGMunAill_c0000002
3300000164_____SI39no09_200mDRAFT_c1000003
3300000436_____LCrCPGB2_illDRAFT_1000001
3300001242_____C687J13896_1000006
3300001242_____C687J13896_1000012
3300001338_____JGI1355J14265_100001
3300001450_____JGI24006J15134_10000002
3300001685_____JGI24024J18818_10000001
3300001729_____JGI24651J20071_1000001
3300001732_____JGI24652J20063_1000001
3300001798_____JGI24126J20157_1000003
3300001798_____JGI24126J20157_1000008
3300001866_____JGI24729J20445_1000001
3300001868_____JGI24146J20443_1000007
3300002071_____JGIcombinedJ21915_10000001
3300002092_____JGI24218J26658_1000009
3300002093_____C687J26627_1000010
3300002294_____B570J29584_1000001
3300002307_____JGI24890J29729_1000009
3300003489_____JGI26540J51217_10000003
3300001685_____JGI24024J18818_10000001

In addition, JGI24723J26617_10000007, a sequence from a dataset analyzed by Sieber *et al.*[22] in our research included a repeated portion that was trimmed and the sequence was retained in the current study.

References

1. Anantharaman, K. *et al.* Thousands of microbial genomes shed light on interconnected biogeochemical processes in an aquifer system. *Nat. Commun.* **7**, 13219 (2016).
2. Borton, M. A. *et al.* Coupled laboratory and field investigations resolve microbial interactions that underpin persistence in hydraulically fractured shales. *Proc. Natl. Acad. Sci. U. S. A.* **115**, E6585–E6594 (2018).
3. Bouma-Gregson, K. *et al.* Impacts of microbial assemblage and environmental conditions on the distribution of anatoxin-a producing cyanobacteria within a river network. *ISME J.* **13**, 1618–1634 (2019).
4. Brown, C. T. *et al.* Unusual biology across a group comprising more than 15% of domain Bacteria. *Nature* **523**, 208–211 (2015).
5. Dalcin Martins, P. *et al.* Viral and metabolic controls on high rates of microbial sulfur and carbon cycling in wetland ecosystems. *Microbiome* **6**, 138 (2018).
6. David, L. A. *et al.* Gut microbial succession follows acute secretory diarrhea in humans. *MBio* **6**, e00381–15 (2015).
7. Devoto, A. E. *et al.* Megaphages infect Prevotella and variants are widespread in gut microbiomes. *Nat Microbiol* (2019). doi:10.1038/s41564-018-0338-9
8. Finstad, K. M. *et al.* Microbial Community Structure and the Persistence of Cyanobacterial Populations in Salt Crusts of the Hyperarid Atacama Desert from Genome-Resolved Metagenomics. *Frontiers in Microbiology* **8**, (2017).
9. Goltsman, D. S. A. *et al.* Metagenomic analysis with strain-level resolution reveals fine-scale variation in the human pregnancy microbiome. *Genome Research* **28**, 1467–1480 (2018).
10. Hug, L. A. *et al.* Aquifer environment selects for microbial species cohorts in sediment and groundwater. *ISME J.* **9**, 1846–1856 (2015).
11. Hug, L. A. *et al.* Critical biogeochemical functions in the subsurface are associated with bacteria from new phyla and little studied lineages. *Environ. Microbiol.* **18**, 159–173 (2016).
12. Kantor, R. S. *et al.* Genome-Resolved Meta-Omics Ties Microbial Dynamics to Process Performance in Biotechnology for Thiocyanate Degradation. *Environ. Sci. Technol.* **51**, 2944–2953 (2017).

13. Karsenti, E. *et al.* A holistic approach to marine eco-systems biology. *PLoS Biol.* **9**, e1001177 (2011).
14. Lavy, A. *et al.* Microbial communities across a hillslope-riparian transect shaped by proximity to the stream, groundwater table, and weathered bedrock. *Ecol. Evol.* **9**, 6869–6900 (2019).
15. Obregon-Tito, A. J. *et al.* Subsistence strategies in traditional societies distinguish gut microbiomes. *Nature Communications* **6**, (2015).
16. Olm, M. R. *et al.* Genome-resolved metagenomics of eukaryotic populations during early colonization of premature infants and in hospital rooms. *Microbiome* **7**, 26 (2019).
17. Probst, A. J. *et al.* Differential depth distribution of microbial function and putative symbionts through sediment-hosted aquifers in the deep terrestrial subsurface. *Nat Microbiol* **3**, 328–336 (2018).
18. Rahman, S. F., Kantor, R. S. & Huddy, R. Genome-resolved metagenomics of a bioremediation system for degradation of thiocyanate in mine water containing suspended solid tailings. (2017).
19. Rampelli, S. *et al.* Metagenome Sequencing of the Hadza Hunter-Gatherer Gut Microbiota. *Curr. Biol.* **25**, 1682–1693 (2015).
20. Roux, S. *et al.* Ecogenomics and potential biogeochemical impacts of globally abundant ocean viruses. *Nature* **537**, 689–693 (2016).
21. Sharrar, A. M. *et al.* Bacterial secondary metabolite biosynthetic potential in soil varies with phylum, depth, and vegetation type. *bioRxiv* 818815 (2019). doi:10.1101/818815
22. Sieber, C. M. K. *et al.* Recovery of genomes from metagenomes via a dereplication, aggregation and scoring strategy. *Nat Microbiol* **3**, 836–843 (2018).
23. Solden, L. M. *et al.* Interspecies cross-feeding orchestrates carbon degradation in the rumen ecosystem. *Nat Microbiol* **3**, 1274–1284 (2018).
24. Tung, J. *et al.* Social networks predict gut microbiome composition in wild baboons. *Elife* **4**, (2015).
25. Youssef, N. H. *et al.* Candidatus Krumholzibacterium zodletonense gen. nov., sp nov, the first representative of the candidate phylum Krumholzibacteriota phyl. nov. recovered from an anoxic sulfidic spring using genome resolved metagenomics. *Systematic and Applied Microbiology* **42**, 85–93 (2019).

26. Brown, C. T., Olm, M. R., Thomas, B. C. & Banfield, J. F. Measurement of bacterial replication rates in microbial communities. *Nat. Biotechnol.* **34**, 1256–1263 (2016).
